# Awake Bruxism Is Unrelated to Smoking, Despite the Different Psychological Status: A Pilot Study

**DOI:** 10.1111/odi.15270

**Published:** 2025-01-30

**Authors:** Ovidiu Ionut Saracutu, Daniele Manfredini, Alessandro Bracci, Marco Ferrari, Edoardo Ferrari Cagidiaco, Anna Colonna, Matteo Pollis

**Affiliations:** ^1^ Department of Medical Biotechnologies, School of Dentistry University of Siena Siena Italy; ^2^ Department of Neurosciences, School of Dentistry University of Padova Padova Italy

**Keywords:** anxiety, awake bruxism, bruxism management, depression, smoking

## Abstract

**Objective:**

The aim of the present study is to get deeper into the complex interplay that might exist between awake bruxism (AB), tobacco smoking, and anxiety and/or depression symptoms in a group of healthy young adults.

**Materials and Methods:**

The study participants were recruited by advertising the investigation at the University of Siena, Siena, Italy. The inclusion criteria were being in good general health, without systemic diseases or oral diseases. People with ongoing medical or dental treatment and those with a history of systemic disease or temporomandibular disorders were excluded. The participants who were included in the study received a questionnaire containing three sections for the assessment of AB behaviors, anxiety and depression symptoms, and the number of cigarettes smoked.

**Results:**

A total of 141 healthy young adults met the inclusion criteria. Different awake masticatory muscle activities showed different strengths of association with anxiety and depression symptoms. However, no relationship was found between the number of smoked cigarettes and the frequency of the various masticatory muscle activities considered.

**Conclusions:**

The result of the present study shows no clear‐cut association between the frequency of self‐reported AB behaviors and smoking in healthy individuals.

## 1 Introduction

More than a decade ago, bruxism was reconceptualized by an international group of experts as a repetitive jaw‐muscle activity with two distinct circadian manifestations: awake bruxism (AB) and sleep bruxism (SB) (Lobbezoo et al. 2013). A subsequent consensus paper defined sleep bruxism as a masticatory muscle activity during sleep that is characterized as rhythmic (phasic) or non‐rhythmic (tonic) and is not a movement disorder or a sleep disorder in otherwise healthy individuals. Conversely, AB was defined as a masticatory muscle activity during wakefulness that is characterized by repetitive or sustained tooth contact and/or by bracing or thrusting of the mandible and is not a movement disorder in otherwise healthy individuals (Lobbezoo et al. 2018). Five years after the 2018 consensus paper, an explanatory note further stated that bruxism cannot be seen as “the” disorder. Still, it can be seen at most as a sign of co‐occurring disease (Manfredini et al. 2023). Thus, in the current state of knowledge, AB is a collective term for a spectrum of masticatory muscle activities occurring during wakefulness (i.e., teeth contact, mandible bracing, teeth clenching, teeth grinding) that requires getting deeper into the associated factors.

Concerning the epidemiology of AB, two systematic reviews indicate a prevalence ranging from 8% to 31% (Manfredini et al. 2013, 2019; Oliveira et al. 2023). The authors of the reviews state that such data must be taken with caution, considering that the researchers, due to the nonexistence of clear diagnostic items for assessing bruxism, adopted different methodologies. Conversely, researchers can now rely on the Standardized Tool for the Assessment of Bruxism (STAB) (Manfredini et al. 2024), the first non‐stackable multidimensional evaluation system of bruxism, developed by an enlarged group of experts compared to the consensus papers (Manfredini et al. 2020, 2024). The STAB contains a series of items related to the evaluation of bruxism status and potential clinical consequences (Axis A) as well as a part assessing the different possible etiological and risk factors (Axis B).

It is well known that psychological factors have an important role in the presence of AB behaviors (Manfredini and Lobbezoo 2009; Colonna and Manfredini 2023). Many studies showed an association between self‐reported AB and psychological conditions, such as anxiety, depression, and stress (Câmara‐Souza et al. 2023; Colonna et al. 2024; Goulart et al. 2021; Manfredini et al. 2005, 2004). Such association was further elucidated from a quantitative point of view through the instrumental assessment of AB behaviors via the ecological momentary assessment (EMA) approach (Saracutu et al. 2024).

Along with psyche, substance abuse, such as tobacco smoking, is also associated with bruxism (Manfredini et al. 2019; Colonna and Manfredini 2023). However, the potential cause‐and‐effect relationship between smoking and bruxism is far from clear. A systematic review on the topic showed that bruxism seems to be twice more prevalent in smokers than in non‐smokers (Bertazzo‐Silveira et al. 2016). An investigation performed on Dutch adolescents suggested that smokers have a 1.42 odds ratio (OR) of having a higher frequency of AB behaviors compared to non‐smokers (van Selms et al. 2013). In contrast to this, a recent retrospective study on a large sample of 8410 Finnish adults showed that smoking cessation is not associated with a decrease in bruxism (Ahlberg et al. 2024).

Smoking is also potentially related to some psychological factors. Smokers report that tobacco smoking helps them relieve stress (Grad et al. 2019; Berlin et al. 2003). On the other hand, chronic smoking is also associated with a higher level of anxiety and depression (Picciotto, Brunzell, and Caldarone 2002; Breslau, Kilbey, and Andreski 1991; Mykletun et al. 2008). Nonetheless, until now, no investigation has tried to evaluate if psyche and tobacco have a synergic effect on the frequency of AB behaviors.

Within these premises, the aim of this study is to get deeper into the complex interplay that might exist between AB, tobacco smoking, and anxiety and/or depression symptoms, in a group of healthy young adults, using a series of items taken from the STAB (Manfredini et al. 2024). Thus, a multiple‐variable model was created to predict the influence of cigarette smoking and psychological distress on the frequency of AB.

## 2 Materials and Methods

### 2.1 Participants Recruitment

The study participants were recruited by advertising the investigation at the University of Siena, Siena, Italy. The inclusion criteria were being in good general health, without systemic diseases or oral diseases. People with ongoing medical or dental treatment and those with a history of systemic disease or temporomandibular disorders (TMDs) were excluded. The TMD Pain screener was administered to rule out TMD patients (Gonzalez et al. 2011). The participants who were included in the study received a questionnaire containing three sections for the assessment of AB behaviors, anxiety and depression symptoms, and the number of cigarettes smoked.

All individuals gave their informed consent in accordance with the Helsinki Declaration and understood that they were free to withdraw from the study at any time. The research protocol was approved by the Institutional Review Board of the Orofacial Pain Unit, University of Siena, Siena, Italy (#0008–2023).

### 2.2 Awake Bruxism Assessment

For the assessment of AB, a series of items from the STAB were adopted. Such questions were originally taken from the Oral Behavior Checklist (OBC) questionnaire (Markiewicz, Ohrbach, and McCall Jr 2006).

Q1: awake grinding question (A2.1 of STAB).

How often do you grind your teeth together during waking hours, based on the last month?

Q2: awake teeth clenching question (A2.2 of STAB).

How often do you clench your teeth together during waking hours, based on the last month?

Q3: awake teeth contact question (A2.3 of STAB).

How often do you press, touch, or hold your teeth together other than while eating (i.e., contact between upper and lower teeth), based on the last month?

Q4: awake mandible bracing question (A2.4 of STAB).

How often do you hold, tighten, or tense your muscles without clenching or bringing teeth together, based on the last month?

For each question the following answer options were provided:

- None of the time.
- A little of the time.
- Some of the time.
- Most of the time.
- All of the time.

Each answer option had the following scoring system based on a 5‐point Likert scale as follows: “none of the time” (0), “a little of the time” (1), “some of the time” (2), “most of the time” (3), “all of the time” (4).

### 2.3 Psychological Assessment

For the psychological assessment, the 4‐item Patient Health Questionnaire‐4 (PHQ‐4) (Item B1.1 of STAB) was adopted. It consists of four questions on anxiety and depression status:

Over the last 2 weeks, how often have you been bothered by the following problems?

- Feeling nervous, anxious, or on edge;
- Not being able to stop or control worrying;
- Feeling down, depressed, or hopeless;
- Little interest or pleasure in doing things.

For each item, the subject is requested to indicate how often they experience each sensation: Not at all = 0, Several days = 1, More than half the days = 2, Nearly every day = 3. The total score can range from 0 to 12 and is rated as normal (0–2), mild (3–5), moderate (6–8), and severe (9–12). A total score ≥ 3 for the first two questions indicates the potential presence of anxiety, while a total score ≥ 3 for the last two questions indicates the potential presence of depression.

### 2.4 Smoking Assessment

For smoking assessment, item B4.3 from the STAB was used.

Do you smoke or use any tobacco products?

- No.
- Yes.
- Quit.

If yes, how many cigarettes/day do you smoke? N°____________.

### 2.5 Statistical Analysis

Data were extracted by two independent reviewers (O.I.S. and M.P.) and put into an Excel document. SPSS 26.0 (SPSS Inc., Chicago, USA) was used to perform the statistical analysis.

Mann–Whitney test was used to assess the differences in the frequency of self‐reported AB and PHQ‐4 between smokers and non‐smokers. The null hypothesis was that the distributions of the variables were equal in the two independent groups. A threshold of *p* < 0.05 was set to reject the null hypothesis. A multiple variable ordinal regression, adjusted for gender and age, was also performed, keeping the frequency of AB as the dependent variable, while psychological distress and the number of smoked cigarettes were considered independent variables. The endpoint data were evaluated using the 95% confidence interval (CI). The level of significance was also set at *p* < 0.05.

## 3 Results

A total of 155 participants were interested in the survey. All of them completed the TMD pain screener, and 14 reported TMD symptoms. Thus, they were excluded from the investigation. After the first screening, 141 subjects met the inclusion criteria for the study. The mean age of participants was 23.34 ± 3.4 years, age range 19–33. Of them, 101 were females, while 40 were males. Table 1 summarizes the results of the AB assessment for all the participants in percentages. Teeth contact and teeth grinding were the less reported AB behaviors. Table 2 indicates the results of the total PHQ‐4. About 35% of the subjects had a PHQ‐4 score higher than normal.

**TABLE 1:** Oral behavior checklist frequencies of the participants.

	None of the time	A little of the time	Some of the time	Most of the time	All of the time
Teeth Contact	42.5% (*n* = 60)	11.34% (*n* = 16)	25.53% (*n* = 36)	12.76% (*n* = 18)	7.80% (*n* = 11)
Mandible Bracing	34% (*n* = 48)	27.66% (*n* = 39)	24.11% (*n* = 34)	13.48% (*n* = 19)	0.71% (*n* = 1)
Teeth Clenching	16.31% (*n* = 23)	32.62% (*n* = 46)	32.62% (*n* = 46)	17.73% (*n* = 25)	0.71% (*n* = 1)
Teeth Grinding	65.96% (*n* = 93)	14.89% (*n* = 21)	15.60% (*n* = 22)	3.55% (*n* = 5)	0% (*n* = 0)

Abbreviation: *n*, Number of participants.

**TABLE 2:** Total PHQ‐4 score of the study population.

Depression and anxiety status	Prevalence (%)
0–2 = normal	65% (*n* = 92)
3–5 = mild	16% (*n* = 22)
6–9 = moderate	18% (*n* = 25)
10–12 = severe	1% (*n* = 2)

Abbreviation: *n*, Number of participants.

Regarding smoking status, 81 of the subjects reported no smoking, and no subjects reported that they quit smoking. The remaining 60 reported smoking cigarettes. The average number of cigarettes smoked per day was 7 ± 4.1, ranging from as low as 1 to as much as 20. Table 3 indicates the different mean frequency of AB and the mean PHQ‐4 between smokers and non‐smokers. The Mann–Whitney U test showed that a statistically significant difference was found between smokers and non‐smokers for the self‐reported frequency of mandible bracing and teeth clenching.

**TABLE 3:** Comparison between PHQ‐4 status and AB frequency between smokers and non‐smokers with the use of Mann Whitney *U* test.

	Non‐smokers *n* = 81 (57%)	Smokers *n* = 60 (43%)	*p*
PHQ‐4 (Mean ± SD)	1.94 ± 2.7	3.4 ± 3.1	0.003*
Teeth Contact (Mean ± SD)	1.5 ± 1.2	2.1 ± 1.4	0.07
Mandible Bracing (Mean ± SD)	1.1 ± 1.0	1.7 ± 1.1	0.021*
Teeth Clenching (Mean ± SD)	1.3 ± 1.1	2.2 ± 0.7	0.0001**
Teeth Grinding (Mean ± SD)	0.4 ± 0.6	0.8 ± 1	0.06

* *p* < 0.5.

** *p* < 0.001.

The results of the multiple ordinal regression analysis unveiled a statistically significant association between the PHQ‐4 scores and the OBC frequency of AB behaviors (Table 4). However, different awake masticatory muscle activities showed different strengths of association with anxiety and depression symptoms. In particular, mandible bracing and teeth clenching showed the strongest association with the PHQ‐4 score with an OR of 1.49 and 1.46, respectively. Teeth contact also showed an OR of 1.33. Conversely, teeth grinding did not show any significant association with the symptoms of anxiety and depression. As a secondary finding, female gender was associated with lower odds of teeth contact (OR = 0.36). Moreover, no relationship was found between the number of smoked cigarettes and the frequency of the various masticatory muscle activities considered (*p* > 0.05) (Table 4).

**TABLE 4:** Results of the multiple ordinal logistic regression analysis.

Dependent variable	Independent variable	Estimate	Odds ratio	Std. error	Sig.	95% confidence interval	
Teeth Contact	Age	0.041	1.04	0.074	0.580	−0.104	0.186
	PHQ‐4	0.286	1.33	0.086	< 0.001**	0.118	0.454
	Number of Cigarettes	0.052	1.05	0.075	0.489	−0.096	0.2
	Smokers	−0.068	0.93	0.545	0.900	−1.136	0.999
	Non‐Smokers	Ref					
	Female	−1.031	0.36	0.418	0.014*	−1.85	−0.212
	Male	Ref					
Mandible Bracing	Age	0.123	1.13	0.076	0.108	−0.027	0.272
	PHQ‐4	0.377	1.46	0.092	< 0.001**	0.196	0.557
	Number of Cigarettes	0.037	1.04	0.078	0.640	−0.116	0.189
	Smokers	−0.348	0.71	0.559	0.533	−1.443	0.747
	Non‐Smokers	Ref					
	Female	−0.114	0.89	0.42	0.787	−0.937	0.71
	Male	Ref					
Teeth Clenching	Age	0.054	1.06	0.076	0.479	−0.095	0.203
	PHQ‐4	0.399	1.49	0.095	< 0.001**	0.214	0.585
	Number of Cigarettes	−0.079	0.92	0.08	0.319	−0.235	0.077
	Smokers	−1.421	0.24	0.571	0.013*	−2.539	−0.302
	Non‐Smokers	0a					
	Female	0.38	1.46	0.418	0.363	−0.439	1.2
	Male	0a					
Teeth Grinding	Age	0.04	1.04	0.082	0.625	−0.12	0.2
	PHQ‐4	0.138	1.15	0.088	0.116	−0.034	0.31
	Number of Cigarettes	0.021	1.02	0.08	0.794	−0.136	0.177
	Smokers	−0.22	0.80	0.592	0.711	−1.381	0.941
	Non‐Smokers	Ref					
	Female	−0.108	0.90	0.455	0.812	−1	0.784
	Male	Ref					

Abbreviation: PHQ‐4, patient health questionnaire‐4.

* Significant at 0.5.

** Significant at 0.001.

## 4 Discussion

The aim of this cross‐sectional investigation was to analyze the relationship between the self‐reported frequencies of AB behaviors with psyche and smoking status in a sample of healthy young individuals. Considering that many systemic or neurological conditions can impact the frequency of AB behaviors, it was decided to include only participants with the absence of systemic diseases (Manfredini et al. 2024). In addition, given the higher frequency of AB among TMD (Câmara‐Souza et al. 2023; Heikkinen et al. 2024) the strong association between psychological distress and TMD (Kurup, Perez‐Pino, and Litt 2024; Manfredini et al. 2024), and the existing triangle of mutual interactions between bruxism–TMD–psyche (Colonna et al. 2024), participants presenting TMD signs and symptoms were excluded, as it could represent an important confounder. Thus, the final study sample was composed of 141 subjects with a mean age of 23.34 ± 3.4 years and an age range between 19 and 33 years. A questionnaire based on some STAB items was administered to evaluate the frequency of AB behaviors, the anxiety and depression symptoms, and the smoking status. For the statistical analysis, a multiple variable model was created. A statistically significant difference was found between smokers and non‐smokers for the self‐reported frequency of mandible bracing (*p* = 0.021) and teeth clenching (*p* = 0.0001). The results of the ordinal logistic regression showed an important association between psychological distress and self‐reported AB. Mandible bracing was strongly correlated with anxiety and depression symptoms (OR = 1.49), followed by teeth clenching (OR = 1.46) and teeth contact (OR = 1.33). However, self‐reported teeth grinding was not associated with the score of the PHQ‐4.

Other studies tried to assess the relationship between AB and smoking, with similar results compared to the present paper. An investigation performed on 2993 Israelian adolescents in 2019 by Winocur et al. showed there is no significant association between AB and smoking. Similarly to the present study, the authors found a strong relationship between psyche and smoking (Winocur et al. 2019). A similar investigation performed on 4235 Dutch adolescents found instead a significant association between the number of smoked cigarettes and the presence of AB with an OR of 1.67 (van Selms et al. 2013). However, it could be argued that both studies were performed on adolescents and that such results cannot be generalized to the whole population. Further studies conducted on patients attending the University clinic were published in the following years. An investigation performed on 50 Brazilian patients aged between 18 and 30 years found no association between smoking and AB (Hilgenberg‐Sydney et al. 2022), while, again, a strong association was found between AB and anxiety. A recent study conducted on 1962 Finnish adults on the association between AB and various conditions found, through a binary regression model, a strong association between AB and psychological distress such as depression and anxiety but no association with smoking (Ekman et al. 2023). Similarly to the present study, all the papers assessed bruxism using a series of items taken from the OBC (Markiewicz, Ohrbach, and McCall Jr 2006).

The main difference is represented by the tool used for the psychological assessment, which, in the case of this paper, was the PHQ‐4. In this regard, the PHQ‐4 showed comparable capabilities to screen anxiety compared to the generalized anxiety disorder 7 (GAD‐7) adopted by Winocur et al. (Winocur et al. 2019). The GAD‐7 has a sensitivity of 0.72 and a specificity of 0.83 (Plummer et al. 2016), which is comparable to the PHQ‐4 (Christodoulaki et al. 2022). In contrast, a recent meta‐analysis found that the state trait anxiety inventory (STAI) adopted by Hilgenberg‐Sydney et al. (Hilgenberg‐Sydney et al. 2022) has a moderate correlation with anxiety (0.59) and depression (0.60) (Knowles and Olatunji 2020). The Hopkins Symptoms Checklist‐25 (HSCL‐25) had a much lower sensitivity for anxiety and depression (43.1%) and a higher specificity (95%) (Mattisson, Bogren, and Horstmann 2013). Nevertheless, no study has ever tried to compare the validity and reliability of the different questionnaires in the same population; thus, a direct comparison of their accuracy with the aim of discussing their impact on results is not possible. Despite this, all the studies found a similar significant association between AB and psyche.

Studies on the association between SB and smoking had similar results. Lavigne et al. hypothesized in their 1997 study, based on the polysomnographic evaluation of SB (Lavigne et al. 1997), that the personality trait might be the real determinant of higher masticatory muscle activity and not necessarily the smoking status. Some years later, the research group of Ahlberg et al. conducted a series of studies on the association between smoking and SB in a sample of Finnish twins that were published. Although a correlation was found between smoking and SB (Rintakoski et al. 2010; Rintakoski and Kaprio 2013), the lack of association was put into question when 10 years later, the data were reviewed (Ahlberg et al. 2024). The revision showed that the SB behavior did not decrease in people who quit smoking. Moreover, as a secondary finding, smokeless tobacco was found to be an independent risk factor for SB.

Based on such findings, it is possible to hypothesize that tobacco itself might not be a real risk factor for bruxism and that the mere act of smoking might be a subconscious behavior adopted by some individuals to relieve stress and anxiety (Choi, Ota, and Watanuki 2015; Parrott 1999), viz., symptoms that have been instead strongly associated with the frequency of AB (Colonna et al. 2024; Goulart et al. 2021; Manfredini et al. 2005, 2005, 2016; Saczuk et al. 2019; Giraki et al. 2010). Both AB (Manfredini et al. 2019) and tobacco smoking (Friedman 2020) could be seen as a coping mechanisms to temporarily relieve stress. If emotional tension has been shown to force subjects to contract the masticatory muscles during stressful situations (Hidaka, Yanagi, and Takada 2004), on the other hand, psychological distress was demonstrated to be significantly more prevalent in current smokers.

It is worth mentioning that the present study was performed on a sample of healthy subjects, while the highest frequencies of AB behaviors have been reported in individuals with specific psychological traits (Montero and Gómez‐Polo 2017), an association that has been confirmed by studies based on the instrumental evaluation of AB (Saracutu et al. 2024). Whether there is a dose–response association between cigarette smoking and AB has yet to be confirmed in studies conducted only on individuals with marked levels of psychological distress.

This study has some limitations. Firstly, a priori sample size calculation was not feasible due to the lack of similar previous studies that could be adopted as a reference, based on the investigation of the association between smoking, as assessed by the number of cigarettes consumed, psychological distress, and AB behaviors. For this reason, this research can be considered a pilot study on this topic, and future works are recommended to deepen the nature of the association between smoking and AB. Secondly, the assessment of bruxism was performed through a questionnaire composed of a series of items taken from the OBC, which are part of the STAB. The accuracy of such items strictly relied on the capability of the participants to recall the frequency of his/her AB behavior frequency. Moreover, the OBC answer options are based on a scale that depends on patients' interpretation, considering that the exact timing is not specified (Grossi and Filho 2024). The third limit is represented by the cross‐sectional design of the study, which could not allow an analysis of the long‐term effect of exposure to cigarette smoking. Another important aspect to consider is the assessment of psychological distress, which was performed through a screening questionnaire without any certified diagnosis. Another aspect to consider is that in the present study, individuals with SB reports, which might be a confounder for data analysis (de Figueiredo, Roithmann, and Grossi 2023) were not excluded. It was demonstrated that the cumulative effect of both SB and AB have very high odds of developing TMD (Sierwald et al. 2015). In the same manner, it could be interesting to study the relationship between SB and AB with smoking, either concurrently or separately. Lastly, all the analyzed variables, such as bruxism frequency, anxiety and depression traits, and smoking status, were all based on self‐report, implying a potential recall bias of the participants involved in the investigation.

The next steps of the research, rather than just performing on larger samples, could be represented by an analysis of the link between bruxism and smoking, thanks to the aid of instrumental devices for the assessment of bruxism. Such analysis could provide evidence of the dose–response relationship between masticatory muscle activity and smoking. In this regard, the EMA represents a cost‐effective tool to assess the frequency of AB behaviors (Câmara‐Souza et al. 2023; Colonna et al. 2020, 2024; Dias et al. 2024). The other option is represented by the surface electromyographic (EMG) devices that permit the quantification of the MMA of the masseter muscle during the 24‐h time span in the natural environment, cutting the cost of expensive laboratory investigations (Colonna et al. 2022, 2024; Anna et al. 2024). In the current state of evidence, the relationship between smoking and bruxism is far from clear.

## 5 Conclusion

The result of the present study shows no clear‐cut association between the frequency of self‐reported AB behaviors and smoking in healthy individuals. Conversely, the frequency of AB behavior was significantly influenced by the severity of the anxiety and depression symptoms.

## Author Contributions

**Ovidiu Ionut Saracutu:** writing – original draft, writing – review and editing, data curation, methodology, formal analysis, investigation, visualization. **Daniele Manfredini:** conceptualization, investigation, methodology, writing – review and editing, formal analysis, supervision, project administration, resources, data curation. **Alessandro Bracci:** conceptualization, investigation, methodology, validation, resources, visualization. **Marco Ferrari:** conceptualization, investigation, validation, visualization, project administration, supervision, resources. **Edoardo Ferrari Cagidiaco:** formal analysis, data curation, validation, visualization, investigation. **Anna Colonna:** writing – review and editing, methodology, investigation, visualization, validation. **Matteo Pollis:** validation, visualization, formal analysis, data curation, writing – review and editing, methodology, software.

## Consent

All individuals gave their informed consent in accordance with the Helsinki Declaration and understood that they were free to withdraw from the study at any time. The research protocol was approved by the Institutional Review Board of the Orofacial Pain Unit, University of Siena, Siena, Italy (#0008–2023).

## Conflicts of Interest

The authors declare no conflicts of interest.

## Data Availability

The data that support the findings of this study are available on request from the corresponding author. The data are not publicly available due to privacy or ethical restrictions.
